# Efficient Node and Sensed Module Management for Multisensory Wireless Sensor Networks

**DOI:** 10.3390/s18072328

**Published:** 2018-07-18

**Authors:** Juan Feng, Xiaozhu Shi

**Affiliations:** 1 School of Aerospace Science and Technology, Xidian University, Xi’an 710071, China; 2 State Key Laboratory of Air Traffic Management System and Technology, The 28th Research Institute of China Electronics Technology Group Corporation, Nanjing 210007, China; xidianshi1998@163.com

**Keywords:** energy efficiency, sensed module management, node selection, WSN

## Abstract

In target tracking wireless sensor networks, choosing a part of sensor nodes to execute tracking tasks and letting the other nodes sleep to save energy are efficient node management strategies. However, at present more and more sensor nodes carry many different types of sensed modules, and the existing researches on node selection are mainly focused on sensor nodes with a single sensed module. Few works involved the management and selection of the sensed modules for sensor nodes which have several multi-mode sensed modules. This work proposes an efficient node and sensed module management strategy, called ENSMM, for multisensory WSNs (wireless sensor networks). ENSMM considers not only node selection, but also the selection of the sensed modules for each node, and then the power management of sensor nodes is performed according to the selection results. Moreover, a joint weighted information utility measurement is proposed to estimate the information utility of the multiple sensed modules in the different nodes. Through extensive and realistic experiments, the results show that, ENSMM outperforms the state-of-the-art approaches by decreasing the energy consumption and prolonging the network lifetime. Meanwhile, it reduces the computational complexity with guaranteeing the tracking accuracy.

## 1. Introduction

Wireless Sensor Networks (WSNs) consist of a large amount of small, low-cost, and wirelessly connected sensor nodes, which have one or more sensed modules, deployed in an unattended natural environment. They have extensive applications, one of which is target tracking [1]. In this application, the user is only interested in the occurrence of a certain event, like movement of an intruder or enemy tanks in battle. These events don’t happen frequently and they commonly have long intervals of inactivity. Specifically, the target tracking scenario can be divided into two stages, namely, surveillance and tracking. During the surveillance state, there is no target of interest in the sensing field, but sensor nodes are ready to detect any possible occurrences. While in tracking stage, the network reacts in response to any moving targets, and collectively tracks and records the roaming path of a moving target.

Since the sensor nodes are usually battery-powered and it is infeasible to replenish energy via replacing their battery after deployment. Therefore, optimization of energy consumption is essential in all aspects of WSN to prolong the network lifetime. In order to save energy, a smaller number of nodes are dynamically chosen for tracking task to balance the energy consumption or reduce the number of work nodes. Hence, it is crucial to select the optimization set of sensor nodes with the minimum cost and quality tracking performance. To solve the node selection problem, the distance-based methods (such as [2,3,4]) are proposed, and they need less computation but cannot reach competitive tracking accuracy. To improve the tracking accuracy, the entropy-based methods (such as [5,6,7]) and the optimal theory-based methods (such as [8,9,10]) are proposed. Although they achieve good tracking accuracy, these methods are computationally expensive. Unfortunately, few works involve the management and selection of the sensed modules for sensor nodes which have several multi-mode sensed modules.

Currently, the researches on the node selection for target tracking are mainly focused on the sensor nodes with a single sensed module. In the existing works, communication module is considered to be the most power-consuming module in a node. The node management mainly refers to turn on all the modules of the selected nodes for target tracking and turn off the communication module or the other modules of the unselected nodes. However, with the development of sensor technology, more and more sensor nodes carry many different types of sensed modules at present, and each sensed module can generate the different sensed signal, such as sound, magnetic, infrared, video and so on [11]. Using multi-mode information to cooperatively complete target tracking tasks is a trend of development in the future, and it can achieve better results than single mode information. For example, the scientists from Intel research team and the University of California, Berkeley, want to track the movements and monitor the habitat of seabirds on Duck Island. Since the seabirds are alert and the environment on Duck Island is very bad, the researchers cannot observe and track the seabirds in a usual way. Therefore, to do this, they apply a self-organizing wireless sensor network, which contains hundreds of sensor nodes equipped with multiple sensed modules, such as light, humidity, acoustic, infrared, camera sensors and so on. These nodes transmit the sensed data to base station computers 300 feet away, via satellite to California servers. However, some of the sensed modules consume a lot of energy. For example, the power consumption of the general video sensor is 20 mW–100 mW, which is larger than the power consumption of the communication module sometimes in sensor nodes. Therefore, it consumes a lot of energy to active all the modules of the sensor nodes. Generally, due to the high density of the sensor nodes, a target always is sensed by many sensor nodes, it is not necessary to collect so much video information. Furthermore, video information generated by video sensed module needs more storage space and larger transmission bandwidth, which leads to consume more energy. Therefore, the selection of sensed modules and the management of their sleep and active state must be taken into consideration.

With these motivations, we propose an efficient node and sensed module management strategy, called ENSMM, for multisensory WSNs. ENSMM dynamically selects the appropriate set of sensed modules and the corresponding nodes to perform tracking tasks, and then the power consumption of nodes is managed according to the selection results. This paper considers the node management in the two different stages, surveillance and tracking. The major objective of ENSMM is to select the best subset of nodes and their sensed modules for the detecting and tracking task with minimum energy consumption. In ENSMM, the models of sensor nodes and sensed modules are more realistic than that in the traditional node management approaches which assume the sensed modules of nodes as one whole module. Furthermore, in ENSMM, each node has multiple sensed modules which have different power consumption and angular diversity of sensor’s sight line. Thus, the sensing ranges of sensors are more realistic than that in the traditional approaches which assume the sensing range of a node as a circle centred on the node. More precisely, the main contributions of this paper include:

- This paper proposes an efficient and adaptive node management strategy, which considers not only node selection, but also the selection of the sensed modules for each node. In the paper, each node has multiple sensed modules and each sensed module has different power consumption and angular diversity of sensor’s sight line. Thus, the nodes can be managed efficiently and the sensor model of the nodes is more reliable and realistic in ENSMM.
- ENSMM selects the node with more residual energy to turn on the sensed module which has higher power consumption. Conversely, the node with less residual energy can be in sleep state or just turn on the lower power consumption sensed modules. Consequently, ENSMM can balance energy consumption with the optimal set of nodes and the corresponding sensed modules so as to prolong the network lifetime.
- This paper proposes an efficient sensed module and node selection algorithm based on a fused decision and detection probability in surveillance stage. Furthermore, in tracking stage, we propose an adaptive sensed modules selection algorithm according to the joint weighted information utility. Therefore, the target can be timely detected and the tracking reliability also is guaranteed with less and balanced energy consumption.
- This paper proposes a joint weighted information utility measurement for multiple sensed modules in different nodes. The joint information utility can be expressed as the overlap area of the sight lines of sensed modules and the covariance-related ellipses. As a result, the complex entropy and the posteriori distribution estimation can be transfer into the simple area calculation to reduce the computational complexity.

## 2. Related Works

Recently, the problem of node management for target tracking in WSNs has been attracting much research attentions. The existing methods are mainly divided into the following three categories: distance-based methods, entropy-based methods and optimal theory-based methods.

For the distance-based methods, the simplest node management strategy is waking up the nodes which are nearest to a target to perform tracking tasks, and let the other nodes in sleep state, such as the method in literature [2,3]. Besides, the authors in [4] propose a weighted distance method based on information measurement. The nodes with minimum weighted distance are chosen to perform the tracking tasks. Although the algorithm also has less computation complexity, it selects only one node each time and does not consider the spatial correlations of nodes. These kinds of methods are simple and easy to implement, but they have low tracking accuracy. To improve tracking accuracy, a combination of distance and utility function is proposed in [12], in which each node extracts a priority value based on its utility function, which is related to the distance between the node and target. The nodes with less priority reduce their sensing range before their neighbors. Then, the nodes that cannot cover any target are not assigned the task and they are turned off. However, the approach requires the location information of all the nodes.

To improve tracking accuracy, the entropy-based node management methods have been proposed. They select the appropriate nodes for tracking tasks depending on the observation information utility of sensor nodes. The authors in [5] propose a heuristic node selection algorithm based on information entropy and implemented by a Bayesian filter. The main idea of the method is to optimize an information utility function using the defined metrics. In [6], the authors propose a mutual-information based sensor selection (MISS) algorithm, which allows the sensor nodes with the highest mutual information about the target state to transfer data first, and the other nodes no longer send their sensed data when the sink received enough data to estimate the target state with the required accuracy. In [7], the authors propose a sensor selection approach based on maximum entropy fuzzy clustering to address the target tracking problem in large-scale sensor networks. They deal with this problem at two levels, sensor-level tracking and global-level fusion. Only a subset of reliable nodes is chosen for track-to-track fusion. In addition, an improved sensor selection approach is proposed for data fusion in both sparse and dense target environments. Although the entropy-based approaches achieve good tracking accuracy, they involve a lot of computations about information entropy and mutual information entropy, and have high computational complexity, especially in the case of a larger number of nodes. In addition, node selections based on information utility function are similar to the entropy-based methods such as those described in [13,14,15]. The main steps of the methods are: (1) building information utility function first according to some specified parameters; (2) and then, selecting nodes to achieve the optimization information utility function. Unfortunately, this kind of methods also has a higher computational complexity.

For the optimal theory-based methods such as [8,10], they establish a linear system of equations whose independent variables are the selected nodes and the constraints are the number of nodes, the energy or the observation range of nodes and so on. The object is to obtain the minimum difference between the observation and actual value so as to get the optimal observation. In [9], the authors propose a novel energy-balanced task-scheduling method for collaborative target tracking using an unscented Kalman filter. At each step of the tracking task, the head node selects active nodes from all nodes within the sensing range to minimize residual energy variations. It is shown a subset selection problem which is NP-hard (non-deterministic polynomial hard), and several energy-balanced scheduling heuristic algorithms are proposed to solve the problem. In [16], the authors propose a probabilistic sensor management scheme based on compressive sensing and probability theory. In the proposed scheme, each node sends its sensing data with a certain probability, the sensor management can be cast as the problem of finding a suitable transmission probability at each node so that a given performance metric is optimized. This method takes the number of selected nodes into account, but it does not consider the residual energy of each node. Also, there are large computational burden. In [17], the authors propose a Fixed-Tree Relaxation-Based Algorithm (FTRA) and a very efficient Iterative Distributed Algorithm (IDA) to obtain the best possible estimation performance at a given querying node. In FTRA, both sensor selection and routing structure are jointly optimized. However, the method selects one sensor node for the tasks at each step and does not consider the locations correlations of the sensing nodes. Literature [18,19] adopt an alternative conditional posterior Cramér-Rao lower bound (C-PCRLB) as the optimization criterion for node selection. Although the total number of participating nodes is limited by a time window, nodes are selected independently without considering the correlation among the observation values of nodes.

In [20], the authors propose an efficient scheduling method based on learning automata, in which a large number of sensor nodes are dispersed randomly in close proximity of a set of targets and the objective of the scheduling mechanism is to select a subset of sensor nodes as active nodes, which can cover all of the targets. And each node is equipped with a learning automaton, which helps the node to select its proper state at any given time. In [21], the authors propose a prolong-stable election protocol (P-SEP) for cluster head elections in the energy-limited heterogeneous fog-supported wireless sensor networks. P-SEP enables uniform nodes distribution, new cluster head selecting policy, and prolongs the time interval of the system, especially before the failure of the first node. Moreover, P-SEP considers two-level nodes’ heterogeneities: advanced and normal nodes, which have the opportunity to become cluster heads. In [22], the authors propose a new method to improve channel assignment and decrease interference in multi-channel wireless mesh networks. The proposed method assigns a channel to each of the links subject to the interface constrains such as minimum amount of network interferences, dynamics of traffic selection for the mesh routers and numbers of hops for assignment so that the implemented channel assignment algorithms are able to adapt themselves to their underlying environment based on their functionalities.

From the above analysis, in the existing work, the node management and selection for target tracking are only for the node which has one sensed module, and if a node is selected, all its modules are simply turned on. Moreover, the existing selection algorithms often have high computational complexity. However, for sensor nodes which have several multi-mode sensed modules, few works involve the management and selection of the sensed modules and how to select the tracking nodes and their corresponding sensed modules.

## 3. System Model

### 3.1. Network Model

We consider a static WSN consisting of one sink and *n* randomly distributed sensor nodes *N_i_*, *i* ∈ [1, *n*], which are deployed uniformly in a two-dimensional sensing field. And *n* is the number of the deployed nodes. The properties of the network model are explained in detail as follows:

- The sink is static and collects the sensing data from the whole sensor network. And it locates far from the network and has an infinite power supply.
- Each node is energy constrained and it is equipped with multiple sensed modules which provide multi-mode sensing data. Then each sensed module has a known sensing range. We assume that sensor node *N_i_* has *m* sensed modules whose sensing data can be express as set *SD* (*N_i_*) = {*M*_1_, *M*_2_, …, *M_m_*}.
- All the sensor nodes are deployed mutually independent and know their positions by using GPS or any localization algorithm. Let *X_i_* (*x_i_*, *y_i_*) be the location of node *N_i_*.

**Figure 1:**
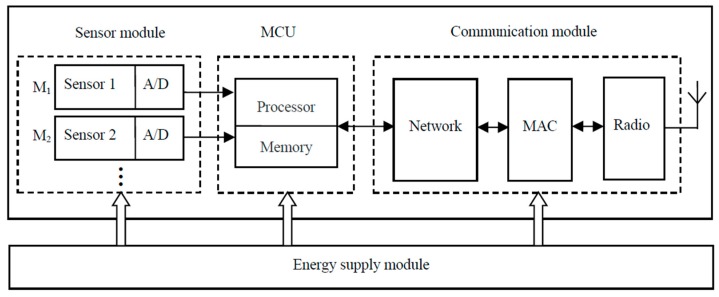
Structure of a sensor node.

Sensor nodes mainly consist of MCU (Microcontroller Unit), communication module, energy supply and sensor components which include several multi-mode sensed modules as shown in Figure 1. These components and modules have three power states, which are active, idle and sleep. Accordingly, sensor nodes have a set of sleep states based on various combinations of component power states to support different levels of power consumption and functionality. Some useful sleep states are listed in Table 1. If one or more sensed modules are active in a node, sensor component is represented as active in Table 1. Then if all the sensed modules are sleep in a node, sensor component is represented as sleep in Table 1. Table 2 detailed the active and sleep states of sensing component.

In the active time slots of the radio, the nodes can receive the assignment messages from the others and check if there are sensing or relaying tasks in the next time instant. If there are tasks, it will remain active; otherwise it will sleep in next sensing instant. In the idle mode of sensor node, its sensing or radio modules remain active but it doesn’t sense, transmit or receive anything so that the node consumes nearly as much power as that in active mode.

The active sensed modules provide the information of the target. Most of the time, not all the sensed modules have to remain active, thus we should select the suitable sensed modules to execute the detecting or tracking task. Because the lifetime of sensor network depends highly on the power consumption performed at each sensor node, we have to make sensor nodes sleep as long as possible. Deeper sleep state makes nodes consume less energy. However, it incurs a longer latency and requires a higher energy cost to awaken. Thus, there is a tradeoff between the energy consumption and the tracking performance.

### 3.2. Target Detection Model

We adopt the weighted target detection mode which utilizes binary assumptions of probability and statistics theory for each sensed module on each node, and then the weighted sum is worked out to indicate the detection result of each sensor node. The target signal detected by each sensed module *j* of each node can be shown as follows:

(1) $$I_{j}(t)=\left\{ \begin{array}{l} g(t),\;\;\;\;S_{0}\\ m_{j}(t)+g(t),\;S_{1} \end{array}\right.,$$

where *g*(*t*) denotes the detection noise which is assumed as white Gaussian noise, *m_j_*(*t*) is the detected signal by sensed module *j* if there is no noise. *S*_1_ and *S*_0_ indicate the target exists and does not at time instant *t*, respectively. If a target occurs in the sensing area of sensor nodes, the detection probability of the sensed module *j* can be calculated as:

(2) $$p_{j}(P_{d}|S_{1})=\int_{\text{Area\_t}}f_{1}(x)dx,$$

where *Area_t* denotes the target existing areas, *f*_1_(*x*) denotes the target detected probability distribution. Similarly, if a target does not occur in the sensing area of sensor nodes, but the sensed module *i* detects the target incorrectly, and the false detection probability can be calculated as:

(3) $$p_{j}(P_{d}|S_{0})=\int_{\text{Area\_u}}f_{0}(x)dx,$$

where *Area_u* denotes the sensing area where the target is not existing, *f*_0_(*x*) denotes the target falsely detected probability distribution. In this case, the sensor nodes are informed about the target appearance and wakened up from the sleep mode. As a result, the sensor nodes consume more energy and detect nothing. In surveillance stage, we should keep the lower false detection probability, while should keep the higher detection probability in tracking stage.

## 4. The Sensor Node Management Algorithm in Surveillance Stage

### 4.1. Management of the Work/Sleep State for Sensed Modules

During surveillance stage, although there is no target in the sensing area, all the sensor nodes should remain at a certain level of vigilance to get ready for detecting. Therefore, the sensed modules of nodes should be awake at the designated time intervals to detect whether there is a target. For multi-sensor nodes, it is energy intensive to keep all the sensed modules working. Also, the working sensed modules generate a lot of sensed data which need to be transmitted through the network. Due to this reason, to avoid missing a target and have less energy consumption, only one or some of the sensed modules should work and the others could have more sleep time.

**Figure 2:**
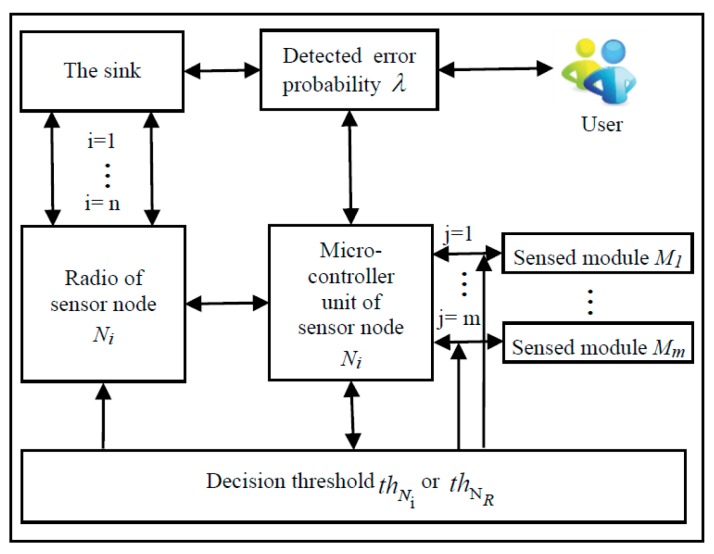
Schematic diagram of sensed modules management

For multi-mode sensor network, a sensor node has more sensed modules and each sensed module has different detected error probability and the different amount of energy consumption. In order to save energy and grantee the detecting performance, we propose the adaptive sensed modules selection strategy based on the detected error probability to select the sensed modules for detecting tasks. The schematic diagram of the proposed strategy shows as Figure 2. First, the sink and all the sensor nodes are informed about the detected error tolerance by the user. Second, the sensor node calculates the detected error probability in the light of the detected error probability of each sensed module and the initial decision threshold, and then it wakes up the sensed modules one by one until the detection accuracy reaches the requirements of the user. At last, the decision threshold is recalculated for the next sensed module selection. In this way, the sleep state of sensed modules is dynamic adjusted to improve the network adaptability and prolong the network lifetime. In addition, each sensed module is assigned different weight on the basis of its detection probability when we calculate decision threshold and detected error probability. The calculations of decision threshold and detected error probability will be described in detail in the following sub-section.

The steps of the sensed modules selection in surveillance stage are detailed as follows:

(1) First, each node sorts its sensed modules from the smallest power consumption module to the biggest one.
(2) Second, node *N_i_* calculates the residual energy level, *RE_L_*(*N_i_*) = [*RE*(*N_i_*)/(*IE*(*N_i_*)/*m*)] + 1, $\left(RE_{L}(N_{i})\in 1,2,\ldots ,m\right)$, where the residual energy level of node *N_i_* is divided into *m* levels and [X] denotes the integral part of X, *RE*(*N_i_*) and *IE*(*N_i_*) are the residual and initial energy of the node *N_i_* respectively.
(3) Then, the node chooses one of its sensed modules to execute the detecting tasks according to its residual energy level and the corresponding power consumption of its sensed modules.
(4) Finally, the node calculates its detected error probability and decision threshold. If the detection accuracy reaches the requirements of the user, the procedure of the sensed modules selection is finished, or else, the node goes to the step (3) and wakes up the sensed module which has smaller power consumption than the last one.

The algorithm of the sensed modules selection in surveillance stage is shown in Algorithm 1. Through the procedure of the sensed modules selection, we can see that the performance of the target detecting meets the requirements of users, and the energy consumption on each sensor node is saved and balanced by just waking up some of the sensed modules.

**Algorithm 1.** Sensed modules selection in surveillance stage.
sensor node = {*N*_1_*…N_n_*}sensed modules = {*M*_1_*…M_m_*} and *p_e_*(*M_j_*)**set***st_j_* = 0, (j∈1,2,…,m)  the initial decision threshold *th_Ni_* = *th_Nr_* = *th_ini_*
**sorting**
  the sensed modules *by their power P_C_(M_j_)*  *P_C_(M_j_) ≤ P_C_(M_j+_*_1_*)* (j∈1,2,…,m)**input** the biggest detected error probability = *λ***for** each $\left(N_{i}\in \{N_{1}\ldots N_{n}\}\right)$  **calculate**    the decision threshold *th_Ni_* or *th_Nr_*    the residual energy level *RE_L_ (N_i_)* $\left(RE_{L}(N_{i})\in 1,2,\ldots ,m\right)$    *RE_L_(N_i_)* = [*RE(N_i_)/(IE(N_i_)/m)*] + 1  **set** *w* = *RE_L_(N_i_)*, *st_w_* = 1  **calculate** *f_Ni_ and f_Nr_*    **if** (*p_e_(N_i_)* > *λ && p_e_(N_r_)* > *λ*)     *W = RE_L_(N_i_)* − 1, *st_w_ =* 1 **end for**


Besides, the node which has more residual energy could wake up the sensed modules which have more power consumption, and vice versa. This way has higher energy efficiency and further improves the network lifetime.

### 4.2. Weighted Detected Error Probability Calculation and Sensed Results Fused Decision Algorithm

**Figure 3:**
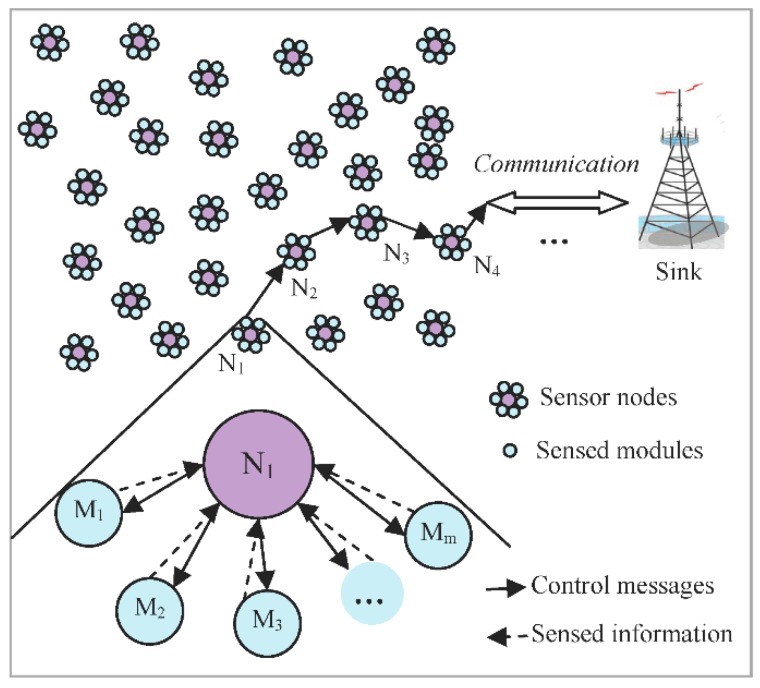
Schematic diagram of the network (Figure 3 is only a sketch map. The number of the sensed modules of the nodes in Figure 3 does not represent the actual number of the sensed modules).

When the procedure of the sensed modules selection is finished, the selected modules start to work and generate the sensed data. The results of the detection are decided by all the sensed information which is fused by the weighted fused decision algorithm. Then, the decision fusion methods about the normal nodes and the relay nodes will be discussed respectively. The normal nodes (such as node *N*_1_ in Figure 3) just have the sensed information from themselves while the relay nodes (such as node *N*_2_ and *N*_3_ in Figure 3) have the sensed information both from their sensed modules and received from other nodes.

In one node, we set up the weighted factor to each sensed module according to its detection probability. For each node, there are *m* sensed modules, and the fused decision is expressed as:

(4) $$f_{N_{i}}=\sum_{j=1}^{m}Z_{d}(M_{j})st_{j}p_{j}(P_{d}|S_{1})(i=1,2\ldots n),$$

where *st_j_* denotes the state of the sensed module, *st_j_* = 1 indicates it is in work state and *st_j_* = 0 indicates the sleep state. $Z_{d}(M_{j})$ denotes the observation of the sensed module *M_j_*. For simplicity in surveillance stage, if the sensed module *M_j_* detects the target $Z_{d}(M_{j})=1$, or else $Z_{d}(M_{j})=0$. Consequently, the decision of the sensor node *N_i_* can be expressed as:

(5) $$d_{N_{i}}=\left\{ \begin{array}{l} 1,\;\sum_{j=1}^{m}Z_{d}(M_{j})st_{j}p_{j}(P_{d}|S_{1})\geq th_{N_{i}}\\ 0,\;\mathrm{otherwise} \end{array}\right.,$$

where $th_{N_{i}}$ denotes the decision threshold of one sensor node.

In the surveillance stage, if one node obtains the detected results according the sensed data from its sensed modules, then the node transmits the results to the sink through the other relay nodes in the network. When the relay node receives the detected information, it will make a fused decision according to both its sensing data from the sensed modules and the received information. Since the detected results received from other nodes are the data fused by the multiple sensed modules, they have a more correct probability than the sensed data come from one sensed module, thus they should acquire the greater weight value. We assume that the received information comes from *n_s_* sensor nodes, and the fused decision of the relay node *N_r_* can be expressed as:

(6) $$f_{N_{r}}=\sum_{j=1}^{m}Z_{d}(M_{j})st_{j}p_{j}(P_{d}|S_{1})+\sum_{i=1}^{n_{s}}p_{N_{i}}d_{N_{i}},$$

where $\sum_{j=1}^{m}Z_{d}(M_{j})st_{j}p_{j}(P_{d}|S_{1})$ denotes the detected results of the relay node self, $\sum_{i=1}^{n_{s}}p_{N_{i}}d_{N_{i}}$ indicates the detected information from the other nodes. $p_{N_{i}}$ represents the weight of the detected information of the node *N_i_*, it can be expressed as:

(7) $$p_{N_{i}}=\sum_{j=1}^{m}st_{j}p_{j}(P_{d}|S_{1})(i=1,2\ldots n_{r}).$$

In this way, the more working sensed modules the node has, the more exact the detected results are, and thus the detected information sent by the node has greater weight value. Therefore, the decision of the sensor node *N_r_* is $d_{N_{r}}=1$ when $f_{N_{r}}\geq th_{N_{r}}$, or else $d_{N_{r}}=0$. In addition, $th_{N_{r}}$ denotes the decision threshold of a relay node.

In the next, we present the calculation algorithms of the decision thresholds in detail. The user can set the biggest detected error probability in the whole network is λ. For one node, the detected error probability *p_e_*(*N_i_*) can be expressed as:

(8) $$p_{e}(N_{i})=p\{\sum_{j=1}^{m}Z_{d}(M_{j})st_{j}p_{j}(P_{d}|S_{1})\geq th_{N_{i}}|S_{0}\}.$$

The detected error probability *p_e_*(*N_i_*) can be calculated based on the central limit theorem and Laplace-DeMoivre approximation as follows [23]:

(9) $$p_{e}(N_{i})\approx Q\left(\frac{th_{N_{i}}-\sum_{j=1}^{m}st_{j}p_{e}(M_{j})}{\sqrt{\sum_{j=1}^{m}st_{j}p_{e}(M_{j})[1-p_{e}(M_{j})]}}\right),$$

where $Q\left(y\right)=\int_{y}^{\infty }(1/\sqrt{2\pi })e^{-t^{2}/2}dt$, $p_{e}(M_{j})$ is the detected error probability of the sensed module *M_j_*. As a result, we can figure out the decision threshold of the node *N_i_* is:

(10) $$th_{N_{i}}\approx \sqrt{\sum_{j=1}^{m}st_{j}p_{e}(M_{j})[1-p_{e}(M_{j})]}Q^{-1}(\lambda )+\sum_{j=1}^{m}st_{j}p_{e}(M_{j}).$$

For the relay node *N_r_*, the detected error probability *p_e_*(*N_r_*) can be expressed as:

(11) $$p_{e}(N_{r})=p\{f_{N_{r}}\geq th_{N_{r}}|S_{0}\}.$$

The relay nodes have to fuse the sensed data from both their own sensed modules and the other sent nodes. Therefore, *p_e_*(*N_r_*) can be further calculated as:

(12) $$p_{e}(N_{r})=\sum_{u=1}^{n_{s}}Q\left(\frac{th_{N_{r}}-\frac{1}{n_{s}}\sum_{i=1}^{n_{s}}p_{N_{i}}d_{N_{i}}-\sum_{j=1}^{m}st_{j}p_{e}(M_{j})}{\sqrt{\sum_{j=1}^{m}st_{j}p_{e}(M_{j})[1-p_{e}(M_{j})]}}\right)\times Q\left(\frac{u-n_{s}\lambda }{\sqrt{n_{s}\lambda (1-\lambda )}}\right),$$

where *u* is the number of the sent node which detected the target, since the biggest detected error probability in the whole network is λ, then $p_{e}(N_{r})=\lambda$. After the network deployed the parameters of *m* and $p_{e}(M_{j})$ are given. Therefore, the above equation has only one unknown variable, which can be figure out by the relay node accord to the received information from other nodes.

## 5. The Sensor Node Management Algorithm in Tracking Stage

When a target occurs in the sensing area, the status of the network is changed from surveillance to tracking. All the nodes whose sensed ranges include the target are regarded as candidate nodes and the corresponding area where the candidate nodes are located forms a tracking area. In tracking stage, the nodes who detect the target report the detected information to the sink, which predicts the position of the target at the next instant and selects some sensor nodes and their sensed modules from the candidate nodes in the tracking area to execute the tracking tasks. When the sink finishes the node and sensed module selections, it will inform the related nodes to change work states. Since the residual energy and the joint weighted information utility of the nodes are considered in our selection procedure, the energy consumption of the network is significantly improved and balanced. Later, when the target moves out of the sensing field, the sink will send message to inform all the nodes to go back to surveillance stage.

### 5.1. Predicting Target Position in View of Particle Filter

In the target tracking application scenarios, due to the sink can obtain the collaborative sensed information of a target, it can efficiently predict the target position in the next sensing instant using PF (particle filter) algorithm, which is a very effective algorithm because it’s potential of coping with difficult nonlinear or non-Gaussian problems. PF with parallel structure bases on Monte Carlo simulation and Bayesian sampling estimation theories [13]. And it is a sequential importance sampling method which is flexible and easy to be implemented.

The steps of PF are outlined as follows:

(1) Initialization

Assuming the initial target position probability distribution is p(X(0)), the particle set is shown as follows:

(13) $$\left\{X_{l}(0),l=1,\cdot \cdot \cdot ,Q_{s}\}\sim p(X(0)\right),$$

where *Q_s_* is the number of particles. *X* (0) is the target position estimation in the initial sensing instant. Thus, $X_{l}(k)=[x_{l}(k),y_{l}(k)]$ is the estimated target position by particle *l*. In addition, the initial importance weight of particle *l* is set as:

(14) $$w_{l}(0)=1/Q_{s}.$$

(2) Iterations

The importance weight of *k* + 1 time instant is calculated as follows:

(15) $$w_{k+1}^{l}=w_{k}^{l}\frac{p(Z_{k+1}|X_{k+1}^{l})p(X_{k+1}^{l}|X_{k}^{l})}{q(X_{k+1}^{l}|X_{k}^{l},Z_{k+1})}=w_{k}^{l}p(Z_{k+1}|X_{k+1}^{l}),$$

where *Z_k+_*_1_ is the observation of target position in the *t* + 1 sensing instant.

(3) Resampling

When the variance of the importance weights becomes excessive, the particle needs to be re-sampled. The effective sample size is defined as:

(16) $$Q_{eff}=\frac{Q_{s}}{1+Var(w_{l}(k))},$$

where *Var* is the variance function. When *Q_eff_* drops below a threshold *Q_th_*, resample *Q_s_* samples according to $p(X_{l}(k)|Z(k))$ and set importance weight of particle *l* as:

(17) $$w_{l}(k)=1/Q_{s}.$$

Thus, the state of target position is updated as:

(18) $$X(k+1)=\sum_{l=1}^{Q_{s}}X_{l}(k+1)w_{l}(k+1).$$

In each sensing instant, the sink node can obtain a prior state of target position for the next sensing instant.

### 5.2. Adaptive Node and Sensed Module Selection

For multi-mode sensor networks, dynamically selecting the best set of sensor nodes and their sensed modules for tracking tasks can reduce the energy consumption of the network and improve tracking accuracy. Furthermore, selecting some of the sensed modules to work and making the others sleep can reduce the amount of sensed information transmitted in the network so that the network energy consumption is further reduced and the network congestion will also be significantly improved. When a target enters into the sensing area, there are many sensor nodes around the target, and one node has multiple sensed modules. However, it is not necessary to track a target with so many sensor nodes and their sensed modules. Generally, we should select sensor nodes and their sensed modules which can bring more information among the candidate nodes until the tracking accuracy reaches the requirements of the user. In addition, the different sensed modules have different performance parameters and power consumption values. To balance the energy consumption, we consider the residual energy of sensor nodes during the sensed modules selection, and choose the nodes that have more residual energy to execute the energy intensive sensory tasks. The sink or the cluster head are responsible for selecting the sensor nodes and their sensed modules.

In the entropy-based method, the entropy is used as information utility measure. The information utility of node *N_i_* is calculated by:

(19) $$Utility(N_{i})=-Hp(X_{t+1}|{\bar{Z}}_{t+1})=\sum P(X_{t+1}|{\bar{Z}}_{t+1})\log P(X_{t+1}|{\bar{Z}}_{t+1}),$$

where $P(X_{t+1}|{\bar{Z}}_{t+1})$ is the posterior distribution of the target’s state, more details of entropy-based information utility measure can be found in [5].

However, the information utility of one multi-sensor node consists of all the sensed information utilities come from each sensed module. The total information utility value of a multi-sensor node is not a simple sum of the information utility value of each single sensed module. In this section, the joint weighted information utility of the sensed modules is calculated in order to select nodes and their sensed module for tracking tasks. To obtain the joint weighted information utility, the information utility of one sensed module is first calculated in more detail below.

**Figure 4:**
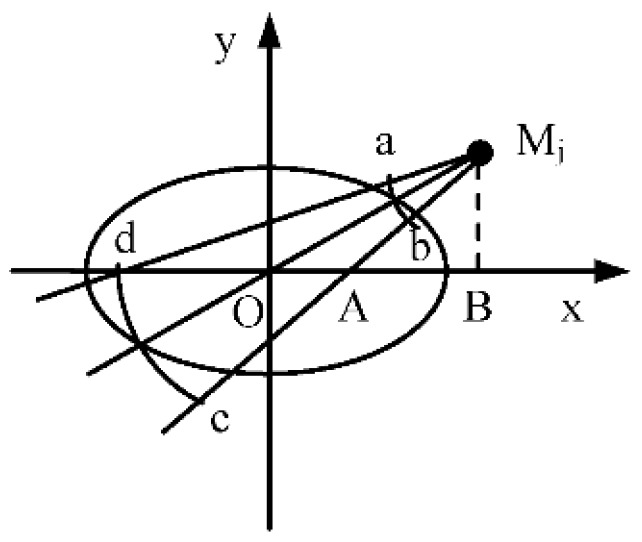
The information utility measure of the sensed module.

The target state predicted by PF algorithm as mentioned in section *A*, from that, we can obtain the predicted location of the target as $\left(\bar{x}\prime ,\bar{y}\prime \right)$ and a covariance matrix as:

(20) $$Cov\prime =\left[\begin{array}{cc} D_{x\prime }^{2} & D_{x\prime }D_{y\prime }r\\ D_{x\prime }D_{y\prime }r & D_{y\prime }^{2} \end{array}\right],$$

where $D_{x\prime }$ and $D_{y\prime }$ are deviations along axes X′ and Y′, respectively, and *r* is the correlation coefficient. Then a new coordinate system, whose origin is at $\left(\bar{x}\prime ,\bar{y}\prime \right)$ and whose axes are along the direction of the eigenvectors of Cov′, can be established. In the new coordinate system, the predicted belief is represented by zero-mean Gaussian density function with covariance:

(21) $$Cov=\left[\begin{array}{cc} \sigma_{x}^{2} & 0\\ 0 & \sigma_{y}^{2} \end{array}\right],$$

where $\sigma_{x}^{2}$ and $\sigma_{y}^{2}$ are the largest and smallest eigenvalue of Cov′, respectively. Then, the state uncertainty of the target can be represented by an ellipse whose major axis and minor axis are $3\sigma_{x}^{2}$ and $3\sigma_{y}^{2}$, respectively as Figure 4 shows. The reason of choosing 3 sigmas is that the state of the target follows a Gaussian distribution within the region covered by a 3σ ellipse and it will appear by the chance of 98.89% [4].

Assuming the measurement error $\Delta \delta_{i}$ is known, and the location $\left(x_{i},y_{i}\right)$ of node $N_{i}$ can be denoted by the polar coordinates $\left(\delta_{i},R_{i}\right)$. Then, the information utility of its sensed module *M_j_* is defined as:

(22) $$Utility(M_{j})=-Area_{abcd}.$$

In this way, the information utility of the sensed module *M_j_* in node *N_i_* can be approximated as the intersecting area of the sight lines of the sensed module and the ellipse, as shown in Figure 4, where the angle of sight lines of sensed module *M_j_* is $2∠OM_{j}A$. The smaller the area is, the more information is provided by the sensed module, and the less state uncertainty of the target is obtained. The equation of the uncertainty ellipse is described as:

(23) $$x^{2}/{\left(3\sigma_{x}\right)}^{2}+y^{2}/{\left(3\sigma_{y}\right)}^{2}=1.$$

**Figure 5:**
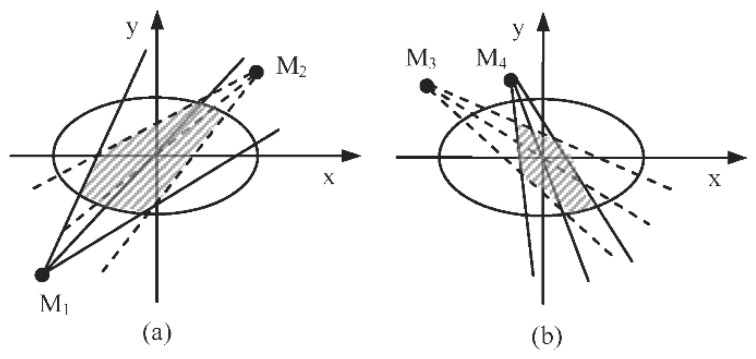
Effection of node’s location in information utility measure: (**a**) the jointed sensing information utility of sensed module *M*_1_ and *M*_2_; (**b**) the jointed sensing information utility of sensed module *M*_3_ and *M*_4_.

The joint weighted information utility of two or more sensed modules are always affected by the position of nodes and the sight line of sensed modules. And the relative position of a target and a senor node affects the information utility of the node. Obviously, when sensor nodes and their sensed modules are selected for tracking tasks, it is inexact to consider the single sensed module each time or to view the joint information utility as a simple sum of the information utility of each module. Taking Figure 5 as an example, the dots *M*_1_, *M*_2_, *M*_3_ and *M*_4_ are four different sensed modules in the different candidate sensing nodes. The joint information utility of *M*_1_ and *M*_2_ are represented as the intersecting area enclosed by the sight lines of *M*_1_, *M*_2_ and the ellipse (the dashed area in Figure 5a). Analogously, the joint information utility of *M*_3_ and *M*_4_ are shown as the dashed area in Figure 5b. To all appearances, the dashed area in Figure 5a is larger than that in Figure 5b. Accordingly, the combination of module *M*_3_ and *M*_4_ can obtain more sensing information utility than that of the module *M*_1_ and *M*_2_. That is to say, choosing module *M*_3_ and *M*_4_ to execute the tracking tasks can obtain more certainty of the target’s state. Therefore, we propose an adaptive node and their sensed modules selection strategy for target tracking based on the joint weighted information utility of some nodes and their sensed modules. That can be calculated as follows:

(24) $$Jo\mathrm{int}\_U(N_{i})=-(w_{1}CA(M_{1})\cap w_{2}CA(M_{2})\cap \ldots \cap w_{j}CA(M_{j})),$$

where *Joint U*(*N_i_*) denotes the joint weighted information utility of node *N_i_*, *CA*(*M_j_*) denotes the certainty area enclosed by the sight lines of module *M_j_* and the ellipse. $w_{1}CA(M_{1})\cap w_{2}CA(M_{2})$ is the weighted overlapped area of the certainty area of module *M*_1_ and *M*_2_. The smaller the area $w_{1}CA(M_{1})\cap w_{2}CA(M_{2})$ is, the more certainty by using the sensing information of module *M*_1_ and *M*_2_ there will be. $w_{j}$ represents the weight of the module *M_j_* and is calculated as follows:

(25) $$w_{j}=st_{j}p_{j}(P_{d}|S_{1}),$$

where *st_j_* = 1/0 denotes the state of *M_j_* work/sleep, $p_{j}(P_{d}|S_{1})$ is the detection probability of *M_j_*. Furthermore, the joint weighted information utility of more candidate sensing nodes can be calculated as follows:

(26) $$Jo\mathrm{int}\_U(N_{1},\ldots ,N_{t})=Jo\mathrm{int}\_U(N_{1})\cap Jo\mathrm{int}\_U(N_{2})\cap \ldots \cap Jo\mathrm{int}\_U(N_{t}),$$

where *t* represents the number of the candidate sensing nodes, $Jo\mathrm{int}\_U(N_{1})\cap Jo\mathrm{int}\_U(N_{2})$ is the jointed sensing information utility of node *N*_1_ and *N*_2_.

To calculate the joint weighted information utility of $w_{1}CA(M_{1})\cap w_{2}CA(M_{2})$ simply, we assume that *Q_p_* points are generated in the ellipse evenly and randomly, then the points fallen in the area of $w_{1}CA(M_{1})\cap w_{2}CA(M_{2})$ are called valid points. The joint weighted information utility of $w_{1}CA(M_{1})\cap w_{2}CA(M_{2})$ can be approximately calculated as the number of the valid points. Similarly, this method can be used to calculate the joint weighted information utility of $Jo\mathrm{int}\_U(N_{1})\cap Jo\mathrm{int}\_U(N_{2})$ or $Jo\mathrm{int}\_U(N_{1},\ldots ,N_{t})$ and so on. In general, we only need to compare the value of two joint weighted information utility without calculating the exactly values in the node selection algorithm. Using the way, the complex entropy calculation is converted to the simple comparing operation. Moreover, we can control the computational complexity and accuracy by choosing proper *Q_p_*.

In our adaptive nodes and their sensed modules selection strategy, the joint weighted information utility measurement is used to effectively select sensor nodes and their sensed modules for tracking tasks. In addition, the residual energy and location of the nodes are considered in the selecting algorithm. The nearest node from the target is preferred and the nodes that have more residual energy are selected to execute the energy intensive sensory tasks. The steps of the sensed modules selection in tracking stage are detailed as follows:

(1) First, each candidate node in the tracking area sorts its sensed modules from the smallest power consumption module to the biggest one and calculates the residual energy level using the same method as Algorithm 1.
(2) Second, each candidate node chooses one of its sensed modules as a candidate tracking module according to its residual energy level and the power consumption of corresponding module. If a node has lower residual energy than residual energy threshold, its candidate tracking module can be withdrawn temporarily and restored in the next round. The residual energy threshold is set as the coefficient *ξ* multiplied by the averaged residual energy of candidate nodes.
(3) Then, the nearest node *N_est_* from the target is selected first and jointed into the tracking tasks. The joint weighted information utility of *N_est_* and each remaining candidate node is calculated separately. The node which has the biggest joint weighted information utility with *N_est_* is selected to join into the tracking tasks.
(4) Then, the target state uncertainty probability *P_un_* is calculated. If the detected target state uncertainty meets the requirements of the user, the procedure of the selection algorithm is finished, or else, it goes to the step (3).
(5) Finally, if every candidate tracking module has been selected and the detected accuracy does not meet the requirements. It goes to the step (2) and each candidate node chooses another candidate tracking module from its sensed modules which has smaller power consumption than last one.

The nodes and their sensed modules which are selected to execute the tracking tasks send their sensed data toward the sink by multi-hop routings. When the sink obtains the data and estimates the position of the target $\left(\bar{x}\prime ,\bar{y}\prime \right)$, then it needs to select and awake the sensor nodes and their sensed modules to track the target for the next time instant. This procedure is continued until the target leaves the deployed sensing area. It can be depicted in Algorithm 2.

**Algorithm 2.** Adaptive nodes and sensed modules selection in tracking stage.
**input** set of candidate nodes *CDD = {N_1_…N_d_}*, d∈[1,D]**input** the biggest tolerance target state uncertainty = *θ*set of selected nodes *SLN*set of selected sensed modules *SLM*set of candidates tracking modules *CDM*set of the nodes included candidate tracking modules *NIM*
*TH_residual_energy = ξAVE(CDD)*
**for** each $\left(N_{i}\in CDD\right)$  **sorting**    the sensed modules *by their power P_C_(M_j_)*    *P_C_ (M_j_)* ≤ *P_C_(M_j+1_)* (j∈1,2,…,m)  **if** (*residual_energy(N_i_)>TH_residual_energy*)    **select** a candidate tracking module *M_C_*    **add** (*M_C_*) to *CDM*    **add** (*N_i_*) to *NIM*  **end if**
**end for**
**add** (*N_est_*) to *SLN***add** (the candidate tracking module of *N_est_*) to *SLM***for** each (*N_s_* ∈NIM−SLN)  **calculate** $Jo\mathrm{int}\_U(SLN,N_{s})$    **if** ($Jo\mathrm{int}\_U(SLN,N_{s})>Jo\mathrm{int}\_U(SLN,N_{t})$,      $N_{t}\in NIM-SLN$ && $N_{t}\neq N_{s}$)     **add** (*N_s_*) to *SLN*     **add** (the candidate tracking module of *N_s_*) to *SLM*   **end if**   **calculate** the target state uncertainty *P_un_*    **if** (*P_un_ > θ*), **goto** line 20
**end for**
**calculate** the target state uncertainty *P_un_*  **if** (*P_un_ > θ*), **goto** line 8**output***SLN* and *SLM*


Suppose the number of the candidate nodes in the tracking area is *Nc*, and each node has *M* sensed modules. Then the computation complexity of ENSMM consists of two parts: (1) selecting sensed modules according to the residual energy of the node, and (2) selecting tracking nodes according to the joint weighted information utility. It is easy to know that the computation complexity of the part 1 is *O*(*Nc*) + *O*(*M*^2^), as selecting sensed modules contains a sort and comparison algorithm. Part 2 can be described as follows: in each iteration, one node is selected from the candidate nodes list such that the joint weighted information utility is optimal, until the target state uncertainty reaches the requirement. The computation complexity of the part 2 is *O*(*Nc*^2^). Since the value of *M* is small, *O*(*M*^2^) has less complexity. Thus, the computation complexity of ENSMM is *O*(*Nc*^2^). In comparison, the computation complexity of the entropy based method is *O*(*Nc*^4^).

## 6. Performance Evaluation

In this section, we illustrate the performance of the proposed node and sensed module management algorithm ENSMM by numerical examples. The simulation is implemented many times in order to find the average results, also the distribution of sensor nodes and the target trajectories are different for each time so that it can avoid the influence of occasionality in one time simulation. The average results of the multiple times are more reliable. However, if the number of simulation times is more than 15, the average results of the simulations tend to stabilize. If we increase the number of simulation times again, there is a little influence on the average value. Therefore, in order to fully compare and verify our proposed algorithms, we conduct experiments under some different network environments, and for each simulation, we run at least 20 times with different random node distributions and the average results are shown. We also compare our simulation results with the distance-based method in [4], the entropy-based method in [6] and the optimal theory-based method in [19] in terms of mean square error, execution time and energy cost and so on.

We consider a WSN, consisting of 200 sensors nodes randomly deployed in a 150 m × 150 m area, and assume each node has an initial energy of 1 J (Joules). The coordinate of the sink is fixed at (0, 0). Each sensor node has three sensed modules, and each sensed module has two statuses, active and sleep, and its energy consumption consists of three parts, working status, transition from active to sleep and transition from sleep to active. The details can be shown as follows:

(27) $$E_{sens}=E_{a-s}+E_{a-w}+E_{acti}=H(e_{a-s}+e_{s-a})+P_{sens}T_{sens},$$

where *H* denotes the number of sensed module turned on or turned off, *P_sens_* and *T_sens_* are the power and working time of the sensed module in active status respectively. Moreover, the measurement error of each sensor follows a Gaussian distribution whose standard deviation is 3 degree, and the other parameters and their values of the sensed modules are summarized in Table 3.

The energy consumption model of the communication module used in this paper is a very widely used model, and described as in Equation (28):

(28) $$\left\{ \begin{array}{l} E_{Tx}(k,d)=(E_{Tx-elec}+\varepsilon_{amp}\times d^{\alpha })\times k\\ E_{Rx}(k)=E_{Rx-elec}\times k \end{array}\right.$$

where *E_Tx−elec_* and *E_Rx−elec_* are the energy consumption of the transmitter and receiver electronics. *ε_amp_* [*Joule/*(*bit*·*m^α^*)] is a constant that represents the energy needed to transmit one bit to achieve an acceptable signal to noise ratio over a distance *d*, and α is the path loss exponent (2≤α≤5) which depends on channel quality. We can assume *E_Tx−elec_* = *E_Rx−elec_* = *E_elec_* and set parameters Eelec = 50 nJ/b, $\varepsilon_{amp}$ = 100 pJ/($b\cdot m^{2}$) and α=2. The bandwidth of wireless channel is 1 Mbps and we adopted the MAC model of IEEE 802.15.4. In PF algorithm, the particle number *N_s_* is set as 500 and the re-sampling threshold *N_th_ =* 0.2 *N_s_*. The sampling period is 1 s. In our work, we set *Q_p_* = 10^3^. The coefficient of the residual energy threshold is set as *ξ* = 0.5. The default value of the biggest detected error probability and the biggest tolerance target state uncertainty are set as *λ* = *θ* = 2%.

The dynamical and state transition model of a target is given as follows:

(29) $$X_{k+1}=\left[\begin{array}{cccc} 1 & T & 0 & 0\\ 0 & 1 & 0 & 0\\ 0 & 0 & 1 & T\\ 0 & 0 & 0 & 1 \end{array}\right]X_{k}+\left[\begin{array}{c} T^{2}/2\\ T\\ T^{2}/2\\ T \end{array}\right]v_{k},$$

where $X_{k}={\left[x,v_{x},y,v_{y}\right]}^{T}$ is the state of the moving target at *k* time instant, *T* is the sampling time interval, *T =* 1 s. x and $v_{x}$ are the position and velocity of the target in the direction of *x* axis respectively. y and $v_{y}$ are the position and velocity of the target in the direction of y axis respectively. $v_{k}$ is the state transition noise of the target, $v_{k}\sim N(0,\sigma )$, and $\sigma =2^{\circ }$. The target in the sensing area moves randomly with a maximum acceleration *a_max_* = 2 m/s^2^ and a maximum velocity *v_max_* = 8 m/s. In addition, the observation model is:

(30) $$Z_{k+1}=HX_{k+1}+w_{k+1},H=\left[\begin{array}{cccc} 1 & 0 & 0 & 0\\ 0 & 1 & 0 & 0\\ 0 & 0 & 1 & 0\\ 0 & 0 & 0 & 1 \end{array}\right],$$

where *H* is the observation matrix, $w_{k+1}$ is the measurement noise which is assumed to be white Gaussian noise sequence with zero means and the variance σ.

Table 4 lists the average energy cost of the nodes, average detected delay and failed detection percentage under different node management algorithms in surveillance stage. In this paper, the average detected delay is defined as the time period between the time when a target enters the deployed area and the time when the sink receives the target information. And the failed detection percentage is defined as the ratio of the number of time which the target was not detected in time to the number of time which the target appeared. We see that ENSMM can obtain lower average detected delay and failed detection percentage with less energy consumption and ENSMM can conserve at least 23% and 28% energy than that in distance-based and entropy-based approaches in the surveillance stage. This is because, both sensor nodes and their sensed modules are selected and managed, and turning off some of the sensing modules not only saves energy but also reduces the number of data stored and transmitted in the network, and then the total energy consumption is less. Besides, the sensed modules are selected based on its detected error probability so as to guarantee the detecting performance. However, as all the sensed modules of a node are managed as an integral unit in the other three methods, so that the energy consumption is higher than that in ENSMM. Although the entropy-based method achieves the lowest failed detection percentage, but it has the longest average detected delay since its node selection algorithm has high computational complexity. In contrast, the distance-based method reduces the computational complexity, which results in a bigger failed detection percentage. Compared with ENSMM, the optimal theory-based method just selects nodes according to the part of the optimal parameters without considering the spatial correlation between the selected nodes, so that it has higher failed detection percentage.

**Figure 6:**
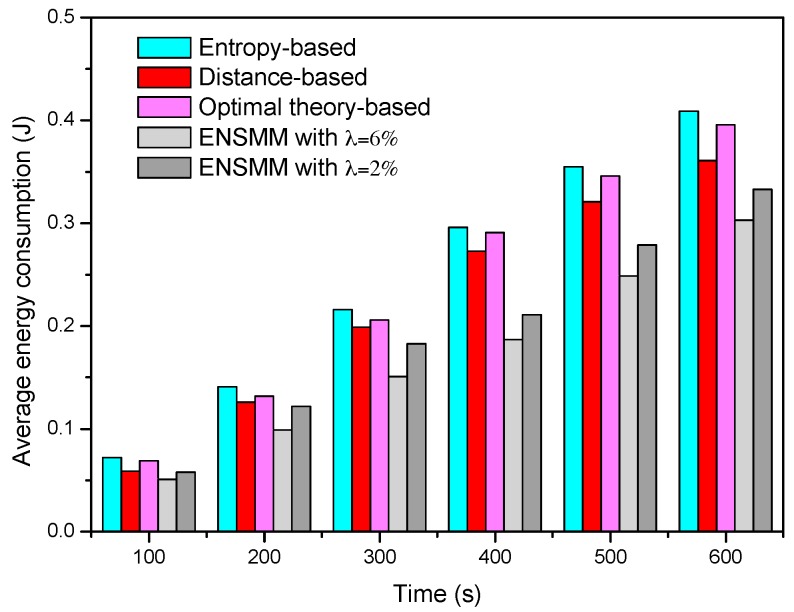
The average energy consumption in surveillance stage.

Figure 6 shows the average energy consumption of each node in surveillance stage under different node management algorithms. We can see that the average energy consumption in ENSMM is lower than that in the other methods. This is because, ENSMM carries out the power management on the sensed modules of each node, and some of sensed modules which have high power consumptions are turned off when no target appears in the deployed area. In addition, the smaller the detected error probability is, the more nodes and sensed modules are required to work, so that the average energy consumption when *λ* = 2% is higher than that in *λ* = 6%. Compared with ENSMM, as all the sensed modules are active when the node is work, the other three methods have higher energy consumption. In the entropy-based method, due to a great deal of calculation on mutual information, it has the highest energy consumption. The optimal theory-based method reduces the computational complexity so as to have lower energy consumption. Nevertheless, the distance-based method has the simplest computation and small computational burden so as to have lower energy consumption than the entropy-based and optimal theory-based method.

**Figure 7:**
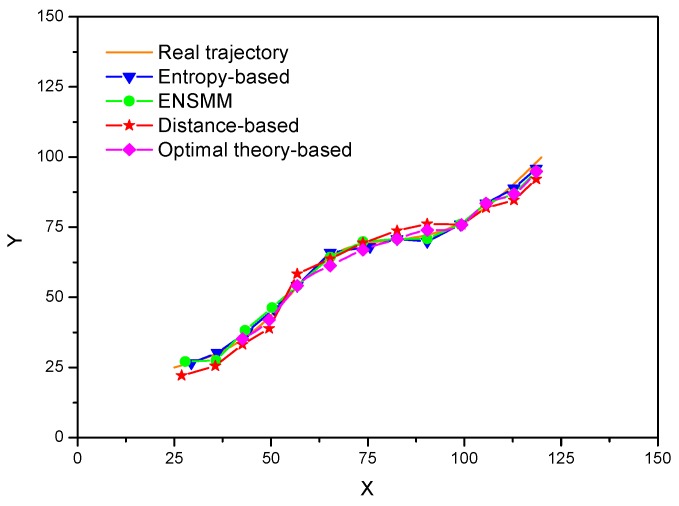
The estimated target trajectory in different methods.

In Figure 7, tracking a target with sensor nodes under the energy constraint is illustrated. Both the true target trajectory and estimated target trajectory are plotted when all the nodes send their measurements to the sink. It is observed that the proposed ENSMM shows similar performance as that of the entropy-based method. This is because the nodes and their sensed modules which can bring the maximum increment of the information utility are selected to meet the tracking accuracy. For the distance-based method, the nodes near to the target are selected by the sink with probability, and without considering the information utility and the correlation of the observations of the nodes, thus the estimated target trajectory has bigger differences with the real trajectory and it is expected to give worse tracking performance compared to the other node management methods.

**Figure 8:**
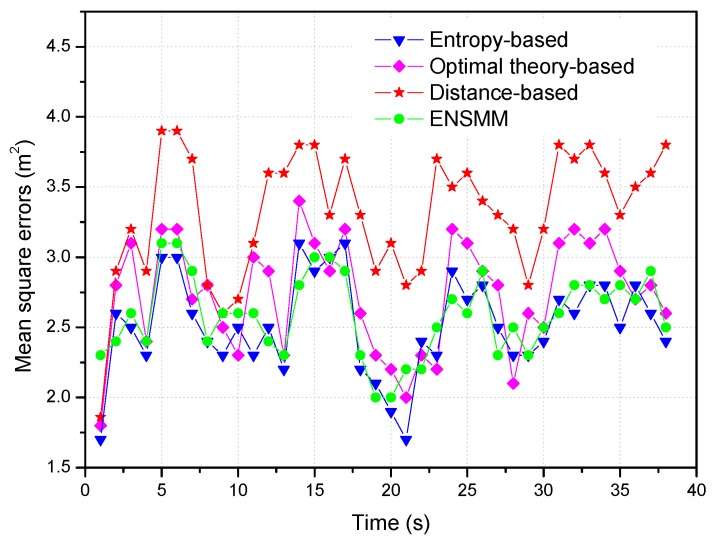
Mean square positioning errors in different methods.

In Figure 8, we show the tracking performance for different node management methods when 3 nodes are selected for tracking. From Figure 8, we can see that ENSMM and the entropy-based method can almost obtain the same performance on the mean square positioning errors. This is because, ENSMM adopts the joint weighted information utility measurement considering the spatial correlation of nodes and their sensed modules, and then it has better chance to activate even more informative nodes and sensed modules for tracking. Likewise, since the entropy-based method executes a lot of probability predictions and intensive entropy calculations to select the appropriate nodes for tracking, so that it has the most accurate results. The optimal theory-based method has less positioning accuracy than ENSMM and entropy-based method because it selects one node each time just to optimize one of the parameters without considering the correlations among the observations of the nodes. For the distance-based method, since it just chooses the nearest sensor nodes from the target to execute the tracking tasks without considering the moving trend of the target and the effect of the angular diversities of sensor nodes, so that it obtains the least positioning accuracy.

**Figure 9:**
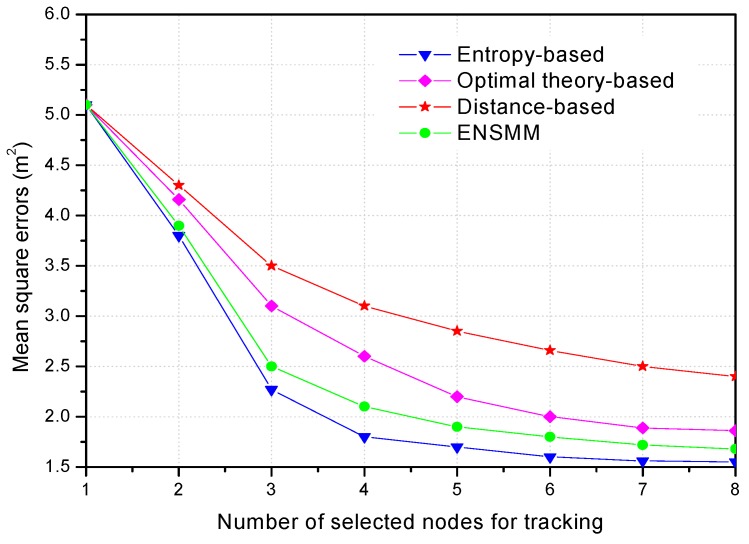
Mean square errors with the different number of selected nodes.

In Figure 9, we show the tracking performance with the different number of selected sensor nodes. Obviously, the more nodes are selected for tracking, the less mean square positioning errors can be obtained, but the network energy consumption is going to increase. When the number of the selected nodes is in the range of 1 to 5, the mean square positioning errors decrease significantly as the number of nodes increases. When the number of the selected nodes is large enough, the tracking performance tends to saturate as shown in Figure 9, and the positioning accuracy increases slowly and inconspicuously because some selected nodes bring the repetitive and useless information. Although the positioning accuracy of the distance-based method rises with the increasing number of selected nodes, it has the worst results because the selected nodes cannot provide informative observations. Besides, ENSMM selects the more informative nodes and sensed modules according to the joint weighted information utility, thus the tracking performance of ENSMM increases substantially when the number of tracking nodes grows from 1 to 5. The optimal theory-based method has worse performance than ENSMM because the correlations of the selected nodes are not considered. Comparing the results of ENSMM with other methods, it is seen that using a fewer nodes and sensed modules, ENSMM can achieve the accuracy which is obtained by selecting more nodes in the optimal theory-based and distance-based methods. Therefore, ENSMM can reduce the number of the active nodes for tracking so as to saving energy and guarantee the tracking performance.

**Figure 10:**
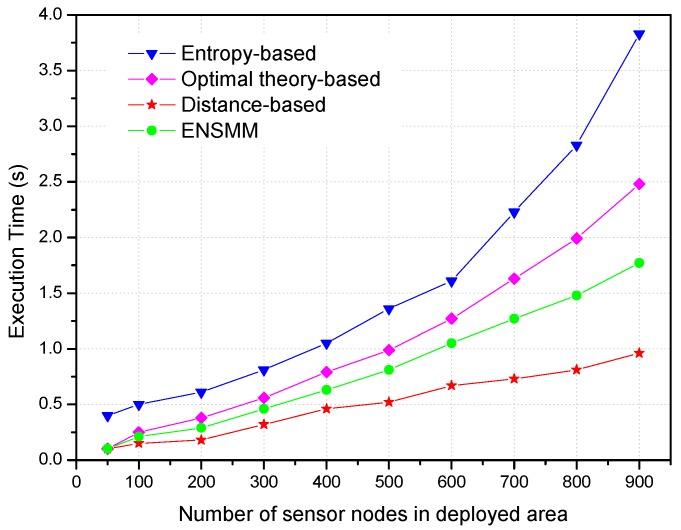
Comparison of execution time versus the number of nodes in deployed area.

In order to investigate that node density in the network affects the computational complexities of these algorithms, the experiments were carried out keeping the other parameters fixed and progressively increasing the number of nodes in the deployed area. Specifically, the number of nodes increases from 50 to 900. We show the effect of the number of nodes on the execution time of the different algorithms in Figure 10. Not surprisingly, all the algorithms use more execution time with the increasing the number of nodes. It is because that there are more candidate nodes in the tracking area, and then more computations are needed for node selection. The execution time of the entropy-based approach quickly rises in pace with the increasing nodes and it is the largest among these methods because more computations of mutual information utilities are needed and it is more complexity with a large number of sensor nodes. The distance-based method uses the shortest execution time compared with the others due to its simple computation. The execution time of ENSMM is shorter than that in the entropy-based and optimal theory-based methods because the complex entropy calculation is converted to the simple area comparing using the proposed joint weighted information utility measurement. From Figure 6 and Figure 7, we can see that ENSMM has relative lower computational complexity to achieve more tracking accuracy.

Moreover, sometimes sensed modules are available with a variable sensing range. If sensing ranges of sensors are changed, the number of candidate nodes within the tracking area is also changed. In a similar way, the longer sensing ranges the sensors have, the more candidate nodes are within the tracking area, and then node selection algorithms need to more computation. Therefore, changing the sensing range of sensors and changing the number of nodes have the similar results.

**Figure 11:**
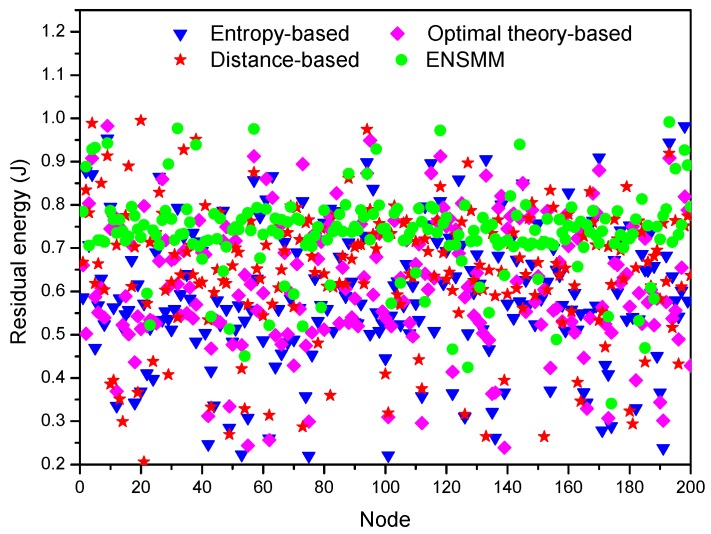
The residual energy of each node under different algorithms.

Now we study the effect of the different algorithms on the energy consumption of each node and the network lifetime. Figure 11 shows the residual energy of each node in the network under different node management algorithms after 300 s simulation time. In all the algorithms, since the nodes close to the target are active and transmitting data frequently so that their energy is quickly depleted and their residual energy is less. However, comparing with the other three algorithms, the residual energy of each node in ENSMM has a concentrated and balanced distribution as shown in Figure 11. This is because the residual energy and power consumption of the sensed modules are considered in ENSMM node and sensed module selection procedure, and then the nodes which have more residual energy are selected to execute the energy intensive sensory tasks, so as to balance the energy consumption. In contrast, the energy consumption of each node is unbalanced in the other three algorithms, and some of the nodes near to the target have a little residual energy and will be exhausted soon.

**Figure 12:**
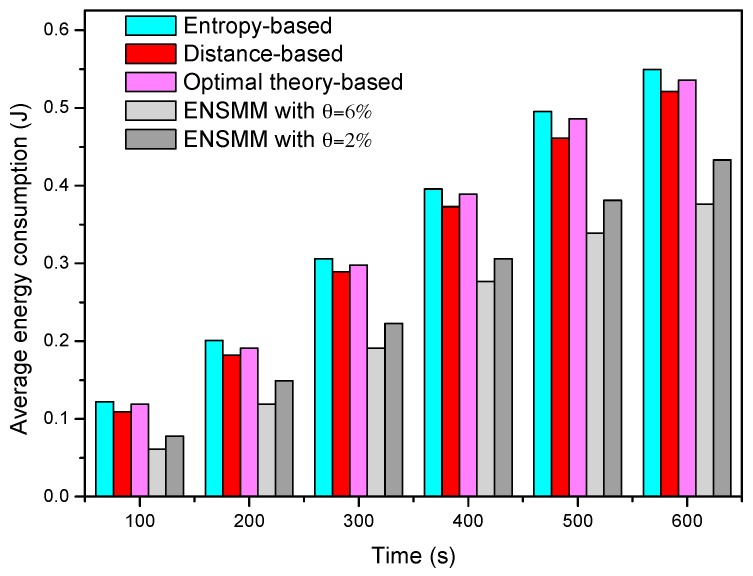
The average energy consumption in tracking stage.

Figure 12 shows the average energy consumption of each node in tracking stage under different node management algorithms. We can see that the average energy consumption is the highest in the entropy-based method. This is because it has the highest computational complexity and when a node is in work status all its sensed modules are active. Although the distance-based and optimal theory-based methods have lower computational complexity, they have not yet managed sleep/work status for sensed modules, so that they also have higher energy consumption. In contrast, the advantages of the proposed ENSMM are even more obvious and the average energy consumption in ENSMM is lower than that in the other methods in the tracking stage. We see that ENSMM can conserve at least 16% and 21% energy than that in distance-based and entropy-based approaches in tracking stage. This is because, ENSMM carries out the power management on the sensed modules of each node, and then not only the sleeping sensed modules can save energy but also the sleeping modules do not transmit sensed data in the network, which will reduce the number of the transmission data and further save energy.

**Figure 13:**
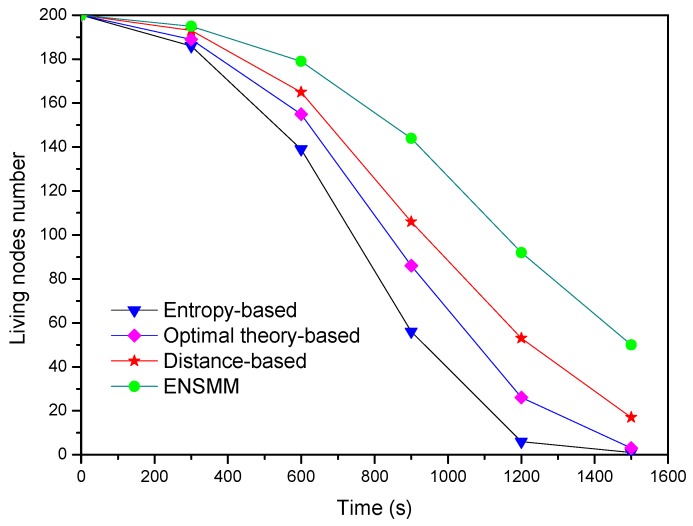
The average energy consumption in tracking stage.

Finally, Figure 13 shows the living node number changes with the simulation time under the different methods. We can see ENSMM has the most living nodes number among these approaches at each time step and the network lifespan in ENSMM is also extended. The main reasons are as following: (1) ENSMM has efficient power management for the nodes and the corresponding sensed modules, so that the idle nodes or sensed modules can be in sleep status to save energy; (2) It has relative less computational complexity. Meanwhile it balances the energy consumption in the network by executing the energy intensive sensory tasks on the nodes which have more residual energy; (3) It reduces the number of the transmitted data by turning off the active sensed modules.

In contrast, the other three methods have unbalanced energy consumption on each sensor node and have not an efficient way to control the sleep/active status for sensed modules. Although the closest approach has the simplest calculations, it needs more active nodes to guarantee the tracking accuracy. The entropy-based approach costs more energy because it has massive calculating works. To sum up, ENSMM achieves more energy efficiency and less computational complexity with degrading the tracking performance.

## 7. Conclusions

In this paper, we propose an efficient node and sensed module management strategy (ENSMM), for multisensory networks. With the proposed approach, the power management of the sensed modules and sensor nodes are efficiently carried out in the surveillance and tracking stages. The major objective of ENSMM is to select the best subset of nodes and their sensed modules for the detecting and tracking task with minimum energy consumption. In surveillance stage, we propose an adaptive sensed modules selection strategy, and each sensed module is assigned different weight on the basis of its detection probability. In addition, we propose a joint weighted measurement to estimate the information utility of the sensor nodes and their sensed modules in the tracking stage so that the appropriate set of sensed modules and the corresponding nodes are dynamically selected to perform the tracking tasks. Numerical results proved ENSMM outperformed the state-of-the-art approaches by reducing the energy cost as well as guaranteeing the tracking performance, for both surveillance and tracking stages.

## Figures and Tables

**Table 1:** Useful sleep states for sensor node.

States	Sensing	Processing	Memory	Radio
st_0_	Active	Active	Active	Rx/Tx
st_1_	Active	Idle	Sleep	Rx
st_2_	Active	Sleep	Sleep	Sleep
st_3_	Sleep	Sleep	Sleep	Sleep

**Table 2:** Sleep states of multi-mode sensed modules.

Sensing	Sensed Module *M*_1_	Sensed Module *M*_2_	Sensed Module *M*_m_
Active	Active	Active	Active
	Active	Active	Sleep
	Active	Sleep	Sleep
Sleep	Sleep	Sleep	Sleep

**Table 3:** The parameters of the different sensed modules.

	Modules	Sensed Module 1	Sensed Module 2	Sensed Module 3
Parameters				
*P_sens_* (mW)		0.1	3	20
*e_a-s_/e_s-a_* (J)		8 × 10^−8^	1.2 × 10^−7^	3.6 × 10^−6^
Sensing range *R_sens_* (m)		15	5	8
Sight angle (degree)		100	30	25
*p_e_* (*M_j_*)		0.2	0.06	0.05

**Table 4:** Comparison of different approaches in surveillance stage.

	Terms	Average Energy Cost of Nodes (J)	Average Detected Delay (s)	Failed Percentage (%)
Approaches				
Entropy-based		0.296	0.83	0.53
Optimal theory-based		0.291	0.56	0.66
Distance-based		0.273	0.36	0.85
ENSMM		0.211	0.45	0.58
